# Corrosion and Mechanical Behavior of the As-Cast and Solid-Solution-Treated AM50 Magnesium Alloy in Different Media

**DOI:** 10.3390/ma16062406

**Published:** 2023-03-17

**Authors:** Miao Yang, Xiaobo Liu, Liyun Xing, Zhaoyu Chen

**Affiliations:** 1 Engineering Training Center, Beihua University, Jilin 132021, China; yangmiao1021@163.com; 2 College of Mechanical Engineering, Beihua University, Jilin 132021, China; 3 College of Electrical and Information Engineering, Beihua University, Jilin 132021, China; 4 Space Environment Simulation Research Infrastructure Harbin Institute of Technology, Harbin 150006, China

**Keywords:** magnesium, solid-solution treatment, corrosion, slow strain rate tensile test

## Abstract

Hydrogen embrittlement and the anodic dissolution mechanism are two important aspects of the corrosion behavior of magnesium alloys. Here, to evaluate the effects of these two aspects on the corrosion failure of magnesium alloys under stress, the stress and corrosion behaviors of the AM50 magnesium alloy in air, deionized water, and NaCl solution after solid-solution (T4) treatment were investigated by X-ray diffraction, scanning electron microscopy, slow strain rate tensile testing, and vacuum dehydrogenation. The as-cast AM50 magnesium alloy was mainly composed of the α-Mg and β-Mg_17_Al_12_ phases. After T4 treatment, the amount of the β-Mg_17_Al_12_ phase was significantly reduced, and only a small amount existed at the grain boundaries. After T4 treatment, the stress corrosion resistance in deionized water improved, but it decreased in an NaCl environment. Dehydrogenation experiments showed that the effect of hydrogen on the corrosion process was weakened owing to the decrease of the β-Mg_17_Al_12_ phase after solution treatment. The effects of hydrogen embrittlement and the anodic dissolution mechanism on the corrosion behavior of the AM50 magnesium alloy under stress were different. In deionized water, the hydrogen embrittlement mechanism played the major role, while the anodic dissolution mechanism played the major role in the presence of Cl^−^ ions.

## 1. Introduction

Magnesium (Mg) alloys are the lightest structural metals with high specific strength and rigidity. Moreover, they are environmentally friendly, biocompatible and convenient for machinery processing [1,2,3,4,5]. Hence, Mg alloys have received widespread attention, and they are often used instead of steels and aluminum in light-weight equipment, the aerospace and automobile industries, and medical applications of orthopedic surgery or cardiovascular medicine [6,7,8]. However, the applications of Mg alloys are limited by the chemical activeness and corrosiveness of Mg [9]. In particular, the corrosion behavior of Mg alloys in a corrosive environment under stress is complex, which calls for further research [10].

In the typical corrosion process of Mg alloys, the cathode and the anode undergo the hydrogen evolution reaction and Mg dissolution, respectively [11]:

The cathode:

2H_2_O + 2e → 2OH^−^ + H_2_

The anode:

Mg − 2e → Mg^2+^

The cathode hydrogen evolution reaction and anode dissolution reaction severely affect the service life and mechanical properties of Mg alloys. Their interactive effect on the service life of materials should be thoroughly investigated, especially when stress and corrosion coexist. Previous research has shown that hydrogen will cause brittle failure in Mg alloys by way of the following four mechanisms: (1) delayed hydride cracking [12,13,14], (2) hydrogen-enhanced localized plasticity [15,16,17], (3) adsorption-induced dislocation emission [18], and (4) hydrogen-enhanced decohesion [16,19,20]. The above research has shown that hydrogen plays a key role in the coupling of stress and corrosion, but there is little research on quantitative assessment of hydrogen embrittlement. The electrode hydrogen charging method has been used to study various hydrogen concentrations [13]. With pre-immersion in a corrosive medium for a certain time, Merson et al. [21] studied stress corrosion cracking of AZ31 and ZK60 Mg alloys by slow strain rate tensile (SSRT) testing. They found that a longer pre-immersion time in the corrosive medium led to more hydrogen embrittlement, and corrosion destruction and hydrogen embrittlement jointly affected the sensitivity to stress corrosion cracking. They also found that if the corrosion products were removed after pre-immersion in the corrosive medium, the effect of hydrogen was significantly alleviated [22]. These studies indicate the crucial role of hydrogen in the stress corrosion behavior of Mg alloys. We believe that cathode hydrogen evolution and anode dissolution during the corrosion of Mg alloys in NaCl solution jointly affect the mechanical behavior of the alloys, but the two effects should be thoroughly investigated and quantified.

In this study, we chose the AM50 Mg alloy and modified the existing form of Al by solution treatment. The stress corrosion behavior of the alloy in air, deionized water, and NaCl solution was then investigated. Through vacuum dehydrogenation, we studied the effects of cathode hydrogen evolution and anode dissolution on the corrosive mechanical behavior after the Mg alloy was pre-immersed in NaCl solution.

## 2. Materials and Methods

### 2.1. Material Preparation

The commercial AM50 magnesium alloy was placed in a graphite crucible and then heated in a 5 kW well furnace (Shenyang electric Furnace Factory, Shenyang, China). When the temperature reached 720 °C, the alloy was cast in a cast-iron mold pre-heated to 200 °C. The cavity size of the mold was 200 mm × 120 mm × 12 mm. Argon was used to protect the molten magnesium alloy from burning. The composition of the alloy was determined by inductively coupled plasma atomic emission spectroscopy (Table 1).

The heat-treated samples were treated by solid-solution treatment (420 °C for 24 h, T4) and were protected by argon gas.

### 2.2. Microstructure Observation

Samples were cut from the same position of the ingot. They were then successively ground with No. 300, 600, 1000, and 1500 waterproof abrasive paper, polished with 1-μm polishing paste, and corroded with 1% nitric acid alcohol.

### 2.3. Corrosive Solutions

Two types of corrosive solutions were selected: deionized water and 3.5% NaCl saturated Mg(OH)_2_ solution. The analytically pure reagents and deionized water were used. First, an NaCl corrosive solution with a mass fraction of 3.5% was prepared. Magnesium hydroxide powder was then added to the solution to form a supersaturated solution, so as to keep the pH of the corrosive solution stable. This solution was used for all of the corrosion experiments involving NaCl solution.

### 2.4. SSRT Test

**Figure 1:**
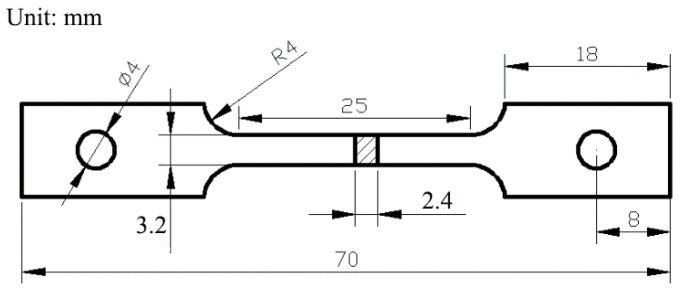
Shape and dimensions of the SSRT samples.

In the SSRT test (RSW10, Changchun Institute of Mechanical Science, Jilin Province, Changchun, China), when the theoretical rate was 1 × 10^−6^ mm/s, the tensile rate was set to 0.0015 mm/min. The shape and dimensions of the SSRT samples are shown in Figure 1. The samples were cut with a wire cutting machine and then drilled with a drilling machine. The samples were connected by pins.

In the case of the corrosion SSRT test, cotton wire winding and drainage were used to keep the corrosive liquid fresh, and it existed in the service area of the samples. The flow rate of the corrosive liquid was approximately 15 drops/min.

During the experiment, three groups of parallel tests were carried out for each state, the average tensile strengths were calculated, and the samples closest to the average value were selected for analysis.

There were two groups of pre-immersed samples. All of the samples were pre-immersed in NaCl solution for 24 h and were then dried. The samples in the first group were immediately SSRT tested in air. The samples in the other group were placed in a vacuum chamber for 30 min when the vacuum reached more than 95%, and they were then sent for SSRT testing.

## 3. Results

### 3.1. Microstructure

**Figure 2:**
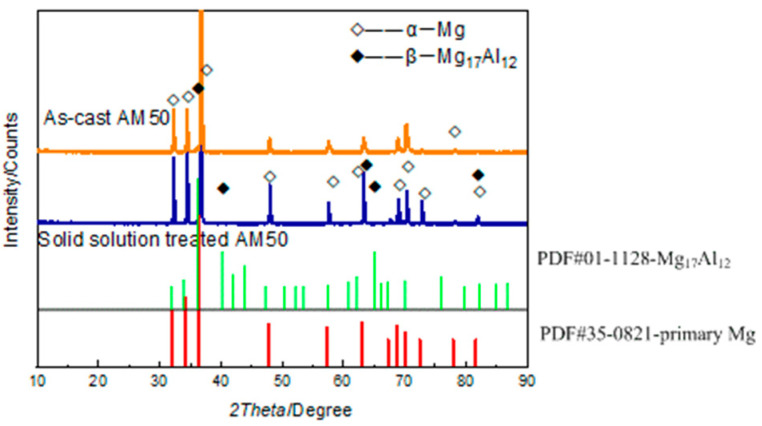
XRD results of the as-cast and solid-solution-treated AM50 magnesium alloy samples.

**Figure 3:**
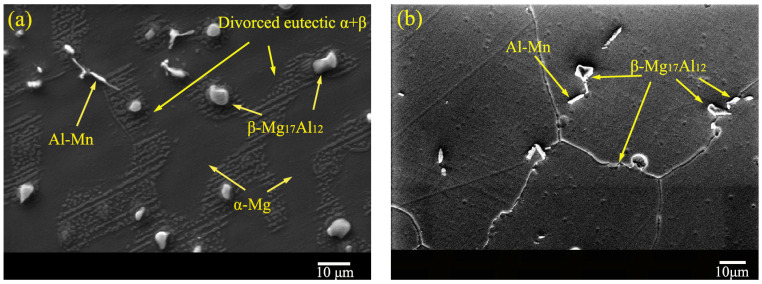
SEM images of the microstructures of the (**a**) as-cast and (**b**) solid-solution-treated AM50 magnesium alloy samples.

The XRD results of the AM50 Mg alloy before and after solid-solution treatment are shown in Figure 2, and the corresponding SEM images are shown in Figure 3. The XRD results showed that both the as-cast and T4-treated alloys consisted of α-Mg and β-Mg_17_Al_12_ phases. They were only different in the peak intensities (Figure 2). The AM50 Mg alloy contained less than 5% Al, the peak of the β-Mg_17_Al1_2_ phase was weaker than that of the α-Mg phase, and the two peaks overlapped. After solution treatment, the amount of the β-Mg_17_Al_12_ phase decreased. Hence, the peak of the β-Mg_17_Al_12_ phase was lower and the peak of the α-Mg phase increased accordingly. The microstructure of the AM50 alloy mainly consisted of the gray island-like β-Mg_17_Al_12_ phase, primary α-Mg phase, and divorced eutectic α + β phase. The β-Mg_17_Al_12_ phase was located at the grain boundary of the α-Mg phase. The average grain size calculated by the straight line method was approximately 53.8 μm. After T4 treatment, the amount of the β-Mg_17_Al_12_ phase in the structure significantly decreased, and only a small amount was left in the grain boundary. In addition, there was a needle-like Al–Mn phase with an average grain size of approximately 55.2 μm.

### 3.2. Corrosion SSRT Test

**Figure 4:**
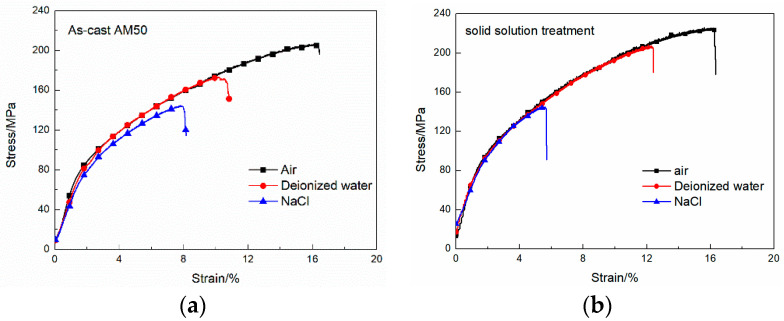
SSRT curves of the (**a**) as-cast and (**b**) solid-solution-treated AM50 magnesium alloy samples in air, deionized water, and 3.5% NaCl aqueous solution.

The SSRT curves of the as-cast and solid-solution-treated AM50 Mg alloy samples in air, deionized water, and 3.5% NaCl solution are shown in Figure 4. The curves showed a similar shape to that of the reported normal stress–strain tensile curves [1,2,7]. They both consisted of a plastic zone and an elastic zone. The elastic zone was short and had no evident transition from the plastic zone, indicating that the fracturing form of the alloys under SSRT conditions was still a quasi-cleavage fracture. For both the as-cast and T4-treated samples, the ultimate tensile strength (UTS) and fracturing elongation (El) determined from the SSRT curves of the alloys in air were the highest. Air, as an inert environment, was used as a reference for subsequent performance analysis by SSRT testing in corrosive media. The UTS and El of the SSRT curves in deionized water both decreased at high strain. The SSRT curves in NaCl solution were the shortest, because both the UTS and El significantly decreased. The overall shapes of the curves were nearly the same, indicating that the material fracture form in deionized water and NaCl solution was the same, although the service strength and time were affected by the environment.

The index of stress corrosion cracking (*I_SCC_*) was calculated using the UTS and El [4]:

(1) $$I_{UTS}=\frac{UTS,environment}{UTS,air}\times 100\%$$

(2) $$I_{El}=\frac{El,environment}{El,air}\times 100\%$$

(3) $$I_{SCC}=iI_{UTS}+iI_{El,}$$

where environment is the corrosive environment (deionized water or 3.5% NaCl solution), air is in air, and *i* is the influence coefficient (here, *i* = 0.5). An overall *I_SCC_* value of closer to 1 indicates that the stress corrosion resistance ability is stronger.

**Figure 5:**
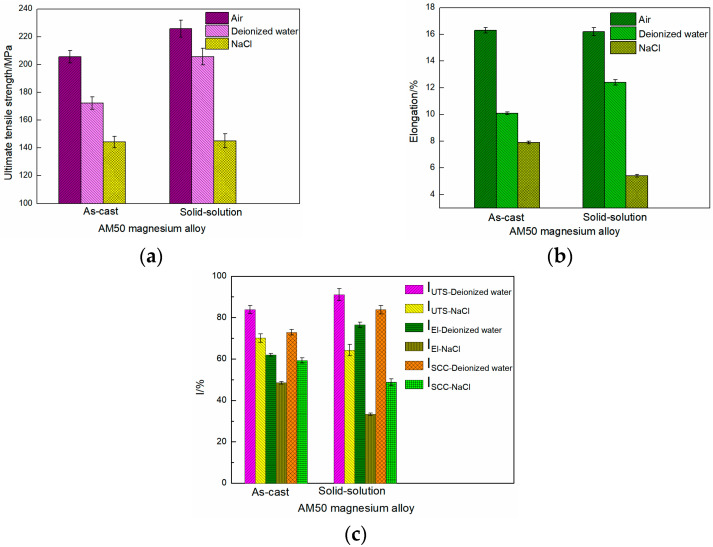
(**a**) UTS, (**b**) El, and (**c**) calculated *I_SCC_* data of the as-cast and solid-solution-treated AM50 magnesium alloy samples.

The UTS and El of the STSS curves before and after thermal treatment are shown in Figure 5a,b, respectively. The UTS and El of the as-cast AM50 Mg alloy were 205.6 ± 4 MPa and 16.3 ± 0.2%, and they were 223.9 ± 6.1 MPa and 16.2 ± 0.3% after solid-solution treatment, respectively. El did not greatly change after solution treatment, but UTS slightly increased. This was mainly because the Mg alloy was a close-packed hexagonal structure and did not greatly deform because of the low content of slip systems at room temperature. After T4 treatment, the β-Mg_17_Al_12_ phase dissolved because the Al element was dissolved in the α phase, which caused lattice distortion and enhanced the solid solution. Thereby, the UTS improved. However, solution treatment did not change the slip system of the Mg alloy, which explained why UTS increased and El did not greatly change.

When deionized water was used as the corrosive medium, the UTS of the as-cast AM50 Mg alloy decreased to 172.3 ± 4.4 MPa and the El was 10.1 ± 0.1%. However, after solid-solution treatment, the UTS and El of the alloy increased to 205.7 ± 6 MPa and 12.4 ± 0.2%, respectively, indicating that solid-solution treatment enhanced the corrosion slow tensile properties of the alloy in deionized water. In 3.5% NaCl solution, the UTS and El of the as-cast AM50 Mg alloy decreased to 144.2 ± 4.2 MPa and 7.9 ± 0.1%, respectively. After solid-solution treatment, they decreased to 145.1 ± 5 MPa and 5.4 ± 0.1%, respectively.

The *I_SCC_* values calculated from the UTS and El data are shown in Figure 5c. In deionized water, *I_UTS_* increased from 83.8 ± 2% (as-cast alloy) to 91.1 ± 2.9% (T4-treated alloy) and *I_El_* increased from 62.0 ± 0.6% (as-cast alloy) to 76.5 ± 1.2% (T4-treated alloy). The overall *I_SCC_* values calculated by Equation (3) were 72.9 ± 1.3% (as-cast alloy) and 83.8 ± 2.05% (T4-treated alloy). In contrast, in NaCl solution, *I_UTS_* decreased from 70.1 ± 2% (as-cast alloy) to 64.3 ± 2.7% (T4-treated alloy), and *I_El_* decreased from 48.5 ± 0.6% (as-cast alloy) to 33.3 ± 0.6% (T4-treated alloy). The overall *I_SCC_* values were 59.3 ± 1.3% (as-cast alloy) and 48.8 ± 1.65% (T4-treated alloy). In general, T4 treatment improved the stress corrosion resistance in deionized water, but it was the opposite in the NaCl solution.

### 3.3. Effect of Dehydrogenation on the Stress Corrosion Sensitivity

To analyze the effects of cathode hydrogen evolution and anode dissolution on stress corrosion, the SSRT samples were pre-immersed in NaCl solution for 24 h. One group was then directly SSRT tested. The other group was dehydrogenated in a vacuum chamber for 30 min. This was because in the corrosion SSRT test the samples fractured after approximately 24 h and hydrogen accumulation and corrosion resistance also occurred after 24 h of immersion, which made the two groups comparable.

**Figure 6:**
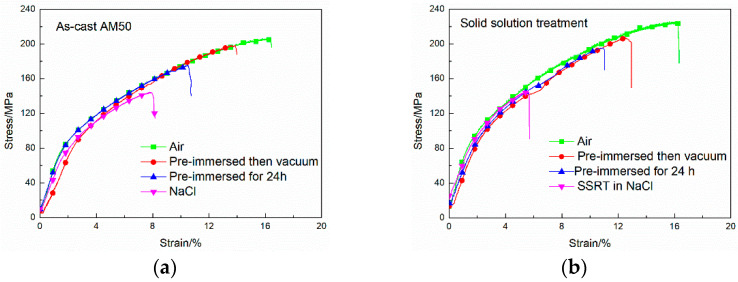
SSRT curves in air, for pre-immersion in 3.5% NaCl solution for 24 h, for vacuum dehydrogenation after pre-immersion in 3.5% NaCl solution for 24 h and in 3.5% NaCl solution of the (**a**) as-cast and (**b**) solid-solution-treated AM50 magnesium alloy samples.

The SSRT curves of the as-cast and solid-solution-treated AM50 Mg alloy samples in air, after immersion in NaCl solution for 24 h and after dehydrogenation, are shown in Figure 6. The UTS results, El results, and calculation data are given in Table 2. The ratios of the differences in the UTS and El values before and after dehydrogenation to the total differences of the UTS and El values before and after immersed corrosion were taken as the influence factors of hydrogen evolution, and they were calculated as follows:

(4) $$I_{H,UTS}=\frac{UTS,hydrogen\;effect}{UTS,total\;effect}=\frac{UTS,vacuum-UTS,immersed}{UTS,air-UTS,immersed}\times 100\%$$

(5) $$I_{H,El}=\frac{El,hydrogen\;effect}{El,total\;effect}=\frac{El,vacuum-El,immersed}{El,air-El,immersed}\times 100\%$$

(6) *I_H,total_* = *iI_H,UTS_* + *iI_H__,__El,_*

where the numerators refer to the hydrogen effect and the denominators refer to the total effect of immersed corrosion. The total effect coefficient *I_H_* was then calculated with the two groups of data using the influence factor of *i* = 0.5.

The UTS and El values of the as-cast and solid-solution-treated AM50 alloy samples after hydrogen removal were both larger than those without hydrogen removal (Figure 6). The UTS and El of the cast AM50 alloy after the SSRT test in air were 205.6 ± 4 MPa and 16.3 ± 0.2%, respectively, and they decreased by 31.0 ± 1.8 MPa to 174.6 ± 4.2 MPa and by 5.9 ± 0.1% to 10.4 ± 0.1%, respectively, after 24 h corrosion in NaCl solution. After hydrogen removal, the UTS and El increased by 23.4 ± 0.2 MPa to 198 ± 4.0 MPa and by 3.5 ± 0% to 13.9 ± 0.1%, which accounted for 75.5 ± 0.1% and 59.3 ± 0% of the total corrosion, respectively. The total effect calculated by Equation (6) was 67.4 ± 0.05%.

The UTS and El of the T4-treated AM50 Mg alloy after SSRT testing in air were 223.9 ± 6.2 MPa and 16.2 ± 0.3%, respectively, and they decreased by 29.7 ± 1.7 MPa to 194.2 ± 4.4 MPa and by 5.3 ± 0.2% to 10.9 ± 0.1% after pre-immersion in NaCl solution for 24 h, respectively. After dehydrogenation treatment, the UTS and El increased by 13.3 ± 0.4 MPa to 207.5 ± 4.8 MPa and by 1.6 ± 0% to 12.5 ± 0.2%, which accounted for 44.8 ± 0.2% and 30.2 ± 0% of the total corrosion, respectively. The total effect calculated by Equation (6) was 37.5 ± 0.1%. Compared with the as-cast sample, on the basis of the UTS, the hydrogen effect of T4 treatment weakened from 75.5 ± 0.1% to 44.8 ± 0.2% by hydrogen removal. On the basis of the El, the hydrogen effect decreased from 59.3 ± 0% to 30.2 ± 0%. The total hydrogen effect decreased from 67.4 ± 0.05% to 37.5 ± 0.1%. These results indicated that, after T4 treatment, the effect of hydrogen during the SSRT process was weakened by pre-immersion in NaCl solution.

The curves of the corrosion SSRT test in NaCl solution in Figure 6 are consistent with those in Figure 4. During the corrosion SSRT test, the UTS and El were both smaller than those in the case of corrosion and then the SSRT test. According to experimental experience, the actual time of corrosion of 24 h was longer than the time of synchronized corrosive tension, which confirms that stress can accelerate corrosion and lead to earlier fracture.

### 3.4. Corrosion Morphologies of the SSRT Samples

**Figure 7:**
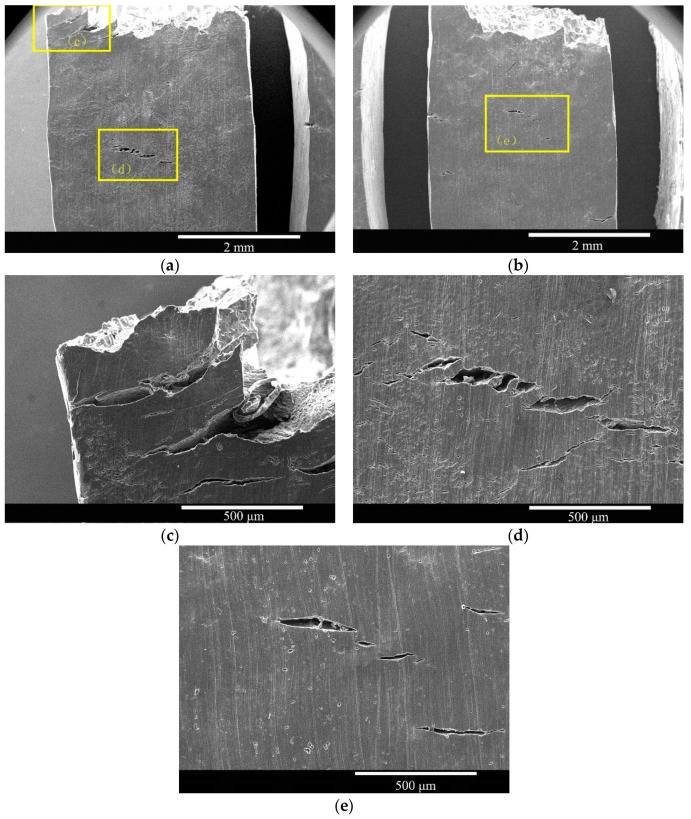
Top-surface morphologies of the (**a**,**c**,**d**) as-cast and (**b**,**e**) solid-solution-treated AM50 magnesium alloy samples corrosion SSRT tested in deionized water.

The top-surface morphologies of the as-cast and solid-solution-treated AM50 magnesium alloy samples SSRT tested in deionized water are shown in Figure 7. The as-cast sample showed two large cracks with a length of 800 μm near the fracture zone (Figure 7c), which were typical torn cracks that nearly penetrated to the lateral side. Moreover, a group of clear cracks with a size of approximately 300 μm was present in the solid-solution-treated sample (Figure 7d). Some corroded areas were also found on the surface. After solid-solution treatment (Figure 7b), the fracture was smooth and the amount of corrosion zones in the middle was clearly less than that in the as-cast sample. Moreover, some cracks formed, including a large crack with size of 300 μm and other smaller cracks (Figure 7e). All the cracks were perpendicular to the tensile direction.

**Figure 8:**
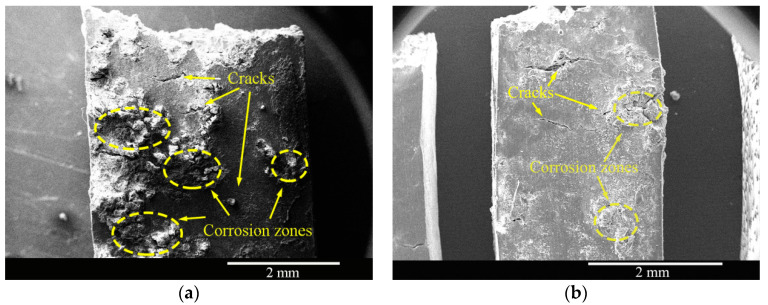
Top-surface morphologies of the (**a**) as-cast and (**b**) solid-solution-treated AM50 magnesium alloy samples corrosion SSRT tested in 3.5% NaCl solution.

The top-surface morphologies of the as-cast and solid-solution-treated AM50 Mg alloy samples SSRT tested in 3.5% NaCl solution are shown in Figure 8. Both of the samples showed corrosion products and cracks on the surface, suggesting that the NaCl solution was far more corrosive and destructive than deionized water and that the cast sample experienced more severe corrosive destruction.

**Figure 9:**
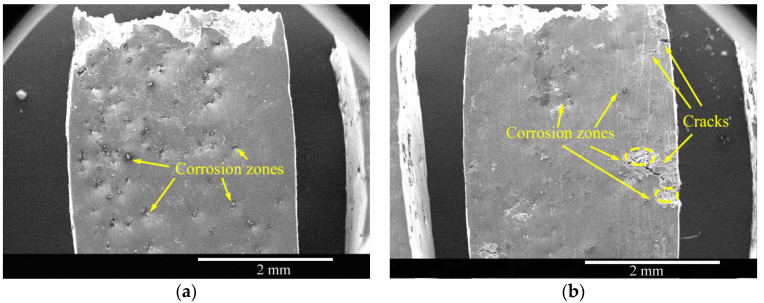
Top-surface morphologies of the (**a**) as-cast and (**b**) solid-solution-treated AM50 magnesium alloy samples SSRT tested after pre-immersion in 3.5% NaCl solution for 24 h.

The top-surface morphologies of the as-cast and solid-solution-treated AM50 Mg alloy samples SSRT tested after pre-immersion in 3.5% NaCl solution for 24 h are shown in Figure 9. Both samples experienced less severe corrosion compared with the corrosion SSRT samples. Using the same experimental method, the amount of surface corrosion zones in the solid-solution-treated sample was smaller than that in the as-cast sample, but one large severely corroded area surrounded by cracks was present in the solid-solution-treated sample.

**Figure 10:**
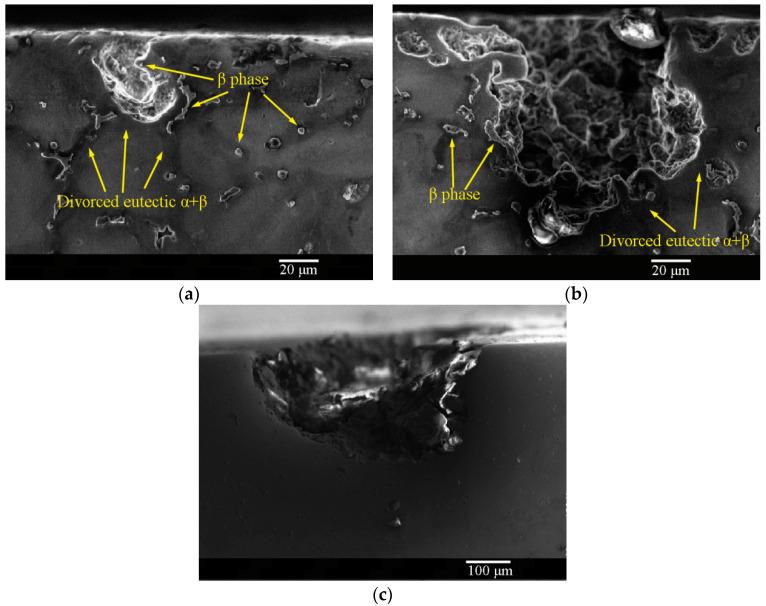
Cross-sectional morphologies of the corrosion pits on the (**a**,**b**) as-cast and (**c**) solid-solution-treated AM50 magnesium alloy samples pre-immersed in 3.5% NaCl solution for 24 h.

Owing to the presence of stress, the number of corrosion pits or the degree of corrosion in the image did not directly reflect the mechanical properties. The morphology of the single corrosion pits and the stress concentration at the bottom of the pits were the direct reasons for fracture. Hence, we investigated the lateral morphology of the corrosion pits. The cross-sectional morphologies of the as-cast and solid-solution-treated AM50 Mg alloy samples after pre-immersion in 3.5% NaCl solution for 24 h are shown in Figure 10. The main corroded part was the preliminary α-Mg phase, and the pit edges were mainly the divorced eutectic α+β phase and β-Mg_17_Al_12_ phase. The depths of the corrosion pits were approximately 30 μm (Figure 10a) or 110 μm (Figure 10b). However, after solid-solution treatment, owing to solution treatment of the β-Mg_17_Al_12_ phase, the boundaries that prevented pit penetration disappeared. Although solid-solution treatment and entrance of Al into the α-Mg phase generally enhanced corrosion, the formation of local single deep pits was sufficient to affect the UTS of the tensile sample. After solid-solution treatment, the depth of the corroded pits increased to 260 μm, which was far larger than that of the as-cast sample. Moreover, the bottom of the corroded pits was sharper and more prone to stress concentration. This result also showed that solid-solution treatment improved the overall corrosion resistance, but it did not enhance the strength of the sample in the corrosion SSRT test.

### 3.5. Fracture Morphologies of the SSRT Samples

**Figure 11:**
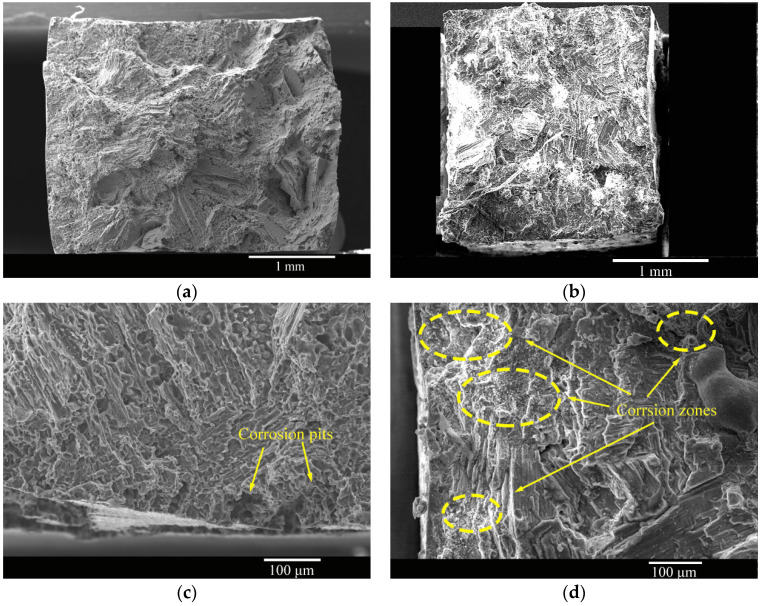
Fracture morphologies of the (**a**,**c**) as-cast and (**b**,**d**) solid-solution-treated AM50 magnesium alloy samples SSRT tested in 3.5% NaCl solution.

The fracture morphologies of the as-cast and solid-solution-treated AM50 Mg alloy samples SSRT tested in 3.5% NaCl solution are shown in Figure 11. The fractures of the as-cast sample were mostly distributed with corrosion traces, and many small, corroded pits were present at the fracture edges. In the fracture morphology of the solid-solution-treated sample, the number of corroded areas at the fractures was smaller than that of the as-cast sample, and no small corrosion pits were present at the fractures. There were some large torn areas, and the corrosion traces were also evident near the edges.

**Figure 12:**
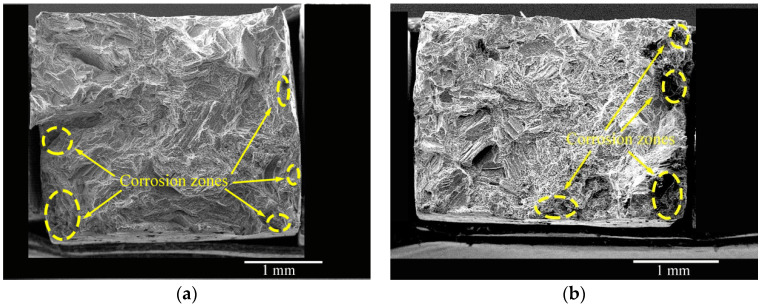

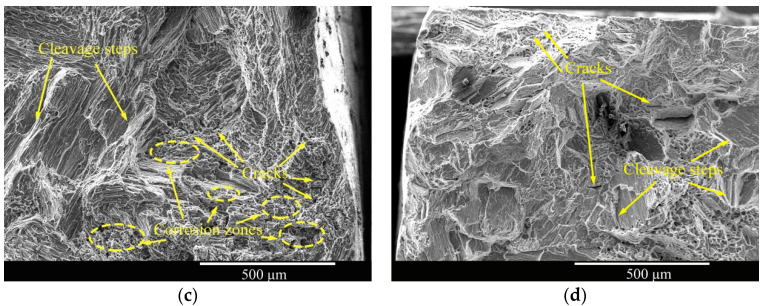
Fracture morphologies of the (**a**,**c**) as-cast and (**b**,**d**) solid-solution-treated AM50 magnesium alloy samples SSRT tested after pre-immersion in 3.5% NaCl solution for 24 h.

The fracture morphologies of the as-cast and solid-solution-treated AM50 Mg alloy samples SSRT tested after pre-immersion in 3.5% NaCl solution for 24 h are shown in Figure 12. Almost no corrosion traces were observed in the middle of both samples. Five corrosion areas existed at the edges of the as-cast sample (Figure 12a). In a magnified image (Figure 12c), cracks were observed in the corroded areas, and river-like patterns and cleavage steps were observed near the middle of the sample. Four large corrosion areas were observed in the solid-solution-treated sample (Figure 12b). Moreover, cracks and smaller cleavage steps were observed. These characteristics indicated that corrosion did not change the mechanism of tensile fracture of the AM50 Mg alloy, which was still quasi-cleavage. From the fracture morphologies, stress concentration at the bottom of surface corrosion pits was the direct reason for fracture failure.

**Figure 13:**
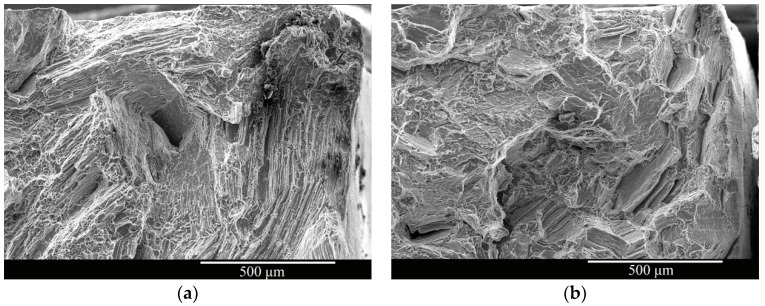
Fracture morphologies of the (**a**) as-cast and (**b**) solid-solution-treated AM50 magnesium alloy samples SSRT tested after pre-immersion in 3.5% NaCl solution for 24 h followed by vacuum dehydrogenation.

The edge fracture morphologies of the as-cast and solid-solution-treated AM50 Mg alloy samples SSRT tested after vacuum dehydrogenation treatment are shown in Figure 13. As with the fracture morphologies of the samples without hydrogen removal, the cleavage steps after solid-solution treatment were smaller than those of the as-cast sample. This was because the solid solution of Al in the α-Mg phase resulted in solid-solution strengthening. Thus, under stress, tearing along the grain boundary was under severe resistance. It then changed to the other direction, which led to a decrease in the size of the cleavage steps.

### 3.6. Al–Mn Phase in the AM50 Magnesium Alloy

**Figure 14:**
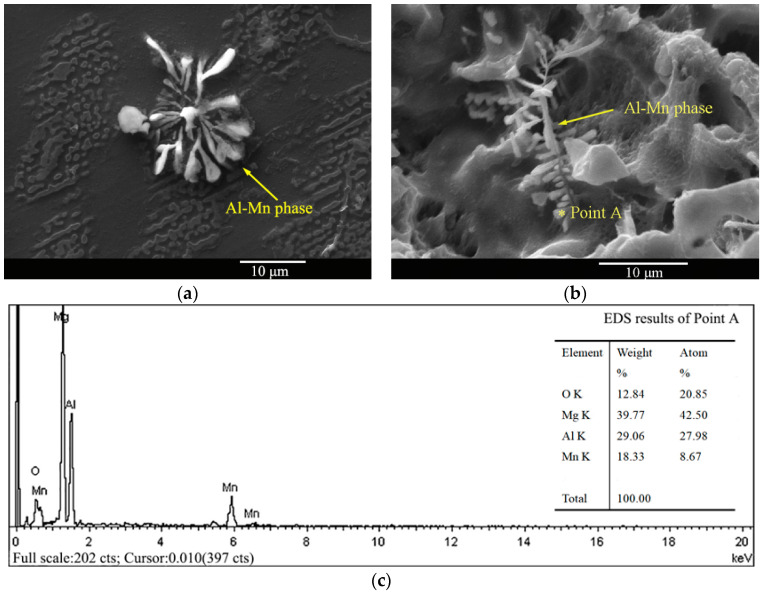
The morphologies of the Al–Mn phase in the as-cast (**a**) and solid-solution-treated (**b**) AM50 Mg alloy samples and the EDS results of point A (**c**).

The morphologies of the Al–Mn phase in the as-cast and solid-solution-treated AM50 Mg alloy samples and the energy-dispersive X-ray spectroscopy (EDS) results of point A are shown in Figure 14. The Mn concentration in the AM50 Mg alloy samples was approximately 0.3%, which can enhance the yield strength and corrosion resistance of the Mg alloy. In an Al-containing Mg alloy, Mn can form the Al–Mn phase in the microstructures. The Al–Mn phases are mainly Al_4_Mn, Al_6_Mn, Al_8_Mn_5_ and Al_11_Mn_4_ [23]. Their shapes are fishbone-, particle-, flower-shape and needle-like because of the different solidification conditions. We found the fishbone-like Al–Mn phase in the as-cast AM50 Mg alloy. Moreover, during corrosion of the α-Mg phase, the Al–Mn phase did not disappear by corrosion, indicating that its corrosion resistance was higher than that of the Mg phase. Using EDS, it was determined that the atomic Al/Mn ratio was close to 3:1. Considering that the basic microstructure might be the β-Mg_17_Al_12_, Al is not entirely present in Al-Mn. Hence, combined with the morphology of the Al-Mn phase, they were most likely Al_8_Mn_5_ [23].

## 4. Discussion

AM50 is a typical Mg–Al alloy, and it is mainly composed of Mg, Al, and Mn. Its structure mainly contains eutectic α-Mg and β-Mg_17_Al_12_ phases. The proportion of Al is approximately 5% (here, it was 4.8687%), and Al mainly enhances the hardness, tensile strength, and casting performance. In addition, the existing form of Al greatly affects the corrosion resistance. In the as-cast AM50 Mg alloy, Al mainly existed in the β-Mg_17_Al_12_ phase together with α-Mg, forming a pair of corrosive micro-galvanic couples, in which β-Mg_17_Al_12_ acted as the cathode to accelerate corrosion of Mg [12]. H separated by the corrosion process is also affected by Al. Existence of the β-Mg_17_Al_12_ phase aids in the storage of hydrogen in Mg alloys [12,24,25]. Thus, after solution treatment, as the content of the β-Mg_17_Al_12_ phase decreases, the hydrogen-storage ability decreases and the effect of hydrogen embrittlement decreases. Our experimental results showed that the effect of hydrogen embrittlement only accounted for 30.2% of the total mechanical loss.

Solution treatment is a common thermal treatment method for Mg alloys. Because the melting point of the β-Mg_17_Al_12_ phase is approximately 420 °C [26], the content of the β-Mg_17_Al_12_ phase significantly decreases and Al is solution-treated into Mg after 24 h of heat treatment. The larger atom size of Al leads to lattice distortion of Mg, which, upon stress action, can pin dislocations. At this moment, the energy needed by deformation increases and the tensile strength is enhanced. Hence, the UTS of the sample can be improved by solution treatment.

Solution treatment of Al also improves the overall corrosion resistance of the alloy. We tested the corrosion resistance of the alloy for 24 h using the weight loss method and found that the weight loss rate of the alloy for solid solution treatment was 3.33 × 10^3^ g·m^−2^·y^−1^, and that of the alloy for gravity casting was 2.45 × 10^3^ g·m^−2^·y^−1^. The improvement of corrosion resistance can also be seen from the corrosion area of the specimen surface. However, the decay rate of mechanical properties under stress conditions cannot be completely determined by the weight loss method. Because corrosion of the AM50 Mg alloy occurs by pitting corrosion, the corrosion-pit distribution and pit morphologies will affect the UTS during the tensile process in two ways: the cross-sectional area of the sample is narrowed owing to corrosion and the stress concentrates at the pit bottom in the case of tension. In the present study, morphological analysis showed that failure was mainly caused by stress concentration at the bottom of the pits. Specifically, after 24-h immersion in NaCl solution, only a few corroded pits had formed on the sample surface, which was far below the degree required to decrease the cross-sectional area. Hence, it was judged that stress concentration at the bottom of the pits and microcrack expansion finally led to fracture failure. This type of failure did not alter the atom arrangement of the crystal cell in the Mg alloy, and the major slip system to undergo deformation was not changed either [27], and was still $\left\{0001\right\}\langle 11\overset{-}{2}0\rangle$. Hence, fracture was still quasi-cleavage fracture, which was confirmed by the shape of the slow tensile curves.

Existing research on the stress corrosion mechanism of Mg alloys acknowledges the presence of two mechanisms: hydrogen embrittlement and anode dissolution [28,29]. Corrosion of the Mg alloy in solution will separate hydrogen from the cathode, which exists as a gas, hydrides, or hydrogen atoms [15]. Hydrogen can easily arrive at the crack tip when stress exists. When the hydrogen concentration at the tip exceeds the local balanced solubility of hydrogen, hydrides will be formed. Brittle hydrides under stress are prone to fracturing, which will repeatedly occur and lead to fracture failure of the alloy [30]. β-Mg_17_Al_12_ in Mg–Al alloys can be the H source of dislocation motion [13,31]. When the Al concentration is 4%–9%, the stress corrosion sensitivity of Mg alloys is high. A larger Al concentration results in the formation of more β-Mg_17_Al_12_ phase and more severe hydrogen embrittlement. In addition, the hydrogen at the crack tip will result in a decrease of the atom binding energy and thereby lower the resistance against dislocation motion, promoting dislocation motion, which causes the material to slip at lower stress [32].

The corrosion of Mg alloys in NaCl solution is mainly determined by the cathode corrosion rate [33], and thus the effect of hydrogen separation should be severe. In the hydrogen removal experiments in the present study, the hydrogen concentration in the alloy was decreased to promote H to form hydrogen gas. The experimental results of the as-cast AM50 Mg alloy showed that the hydrogen embrittlement effect accounted for 67.4 ± 0.05% of the total corrosion. After solid-solution treatment, the effect of hydrogen embrittlement decreased to 37.5 ± 0.1%. This is because solid-solution treatment decreased the amount of the β-Mg_17_Al_12_ phase in the corrosion galvanic couple and weakened the hydrogen-storage ability, improving the corrosion resistance of the alloy. Consequently, the effect of hydrogen embrittlement was less severe.

During electrochemical corrosion of Mg alloys, an anode reaction will also occur to form Mg^2+^ and an oxide/hydroxide layer [34]. Although the corrosion film is not complete or dense, it can still cover the material’s surface and thereby prevent subsequent corrosion. When Cl^−^ exists in the solution, because of its small ionic radius, Cl^−^ can easily pass through the loose corroded film to reach the metal surface, leading to the nucleation of corrosion pits. At this moment, in the presence of stress, the stress concentrates at the bottom of the pits, leading to large avulsion of the material’s surface, and thus the product film is destroyed to expose the new substrate. The resulting corrosion products are sufficient to form a “large cathode”, but the newly exposed substrate is small, so the corrosion current suddenly increases and the crack tip cannot be passivated, which promotes further dissolution in the anode [28]. Thus, the presence of Cl^−^ promotes anode dissolution of Mg alloys under stress. After solution treatment, although the total corrosion resistance of the alloy is enhanced, when stress exists, the formation of only one deep corrosion pit owing to stress concentration will result in rapid fracturing. The improvement of the total corrosion resistance is not active in prolonging the mechanical life, so the material failure is random. Overall, in deionized water without Cl^−^, the thick corrosion film blocks contact between the substrate and liquid, and thus hydrogen evolution corrosion is the major factor influencing corrosion cracking of the material. When Cl^−^ exists, the anode dissolution mechanism is dominant.

## 5. Conclusions

The stress and corrosion behaviors of the AM50 Mg alloy before and after solid-solution treatment have been investigated in air, deionized water, and NaCl solution. The effects of hydrogen embrittlement and anode dissolution on stress corrosion were quantified through a vacuum hydrogen-removal method. The conclusions are as follows:

(1) The gravity-cast AM50 Mg alloy was composed of α-Mg and β-Mg_17_Al_12_ phases. The amount of the β-Mg_17_Al_12_ phase significantly decreased after solution treatment, and only a small amount of the β-Mg_17_Al_12_ phase existed in the grain boundary. The Al–Mn phase in the structure was fishbone-shaped, and it was most likely Al_3_Mn;
(2) After solid-solution treatment, the stress corrosion resistance of the AM50 Mg alloy was enhanced in deionized water, but it was weaker in the NaCl solution;
(3) Dehydrogenation experiments showed that the effect of hydrogen on the corrosion process was weakened owing to the decrease of the amount of the β-Mg_17_Al_12_ phase after solution treatment. Hydrogen removal experiments showed that, after thermal treatment, the effect of hydrogen after pre-immersion in NaCl solution decreased from 67.4 ± 0.05% to 37.5 ± 0.1% during the slow tensile process;
(4) The effects of hydrogen embrittlement and the anodic dissolution mechanism on the corrosion behavior of the AM50 magnesium alloy under stress were different. In deionized water, the hydrogen embrittlement mechanism played the major role, while the anodic dissolution mechanism played the major role in the presence of Cl^−^ ions.

## Figures and Tables

**Table 1:** Composition of the as-cast AM50 magnesium alloy (wt.%).

Al	Mn	Zn	Si	Cu	Ni	Mg
4.8687	0.2823	0.1232	0.0161	0.0015	0.0008	Bal.

**Table 2:** UTS and El data of the SSRT curves under different conditions in Figure 6.

Alloy	Method					Analysis		
	Data	Air	Immersion	Vacuum Dehydrogen	Total Effect	Hydrogen Effect	*I_H_* (%)	*I_H,total_* (%)
As-cast	UTS (MPa)	205.6	174.6	198.0	−31.0	+23.4	75.5	67.4 ± 0.05
	UTS-Error	±6.0	±4.2	±4.0	±1.8	±0.2	±0.1	
	EL (%)	16.3	10.4	13.9	−5.9	+3.5	59.3	
	El-Error	±0.2	±0.1	±0.1	±0.1	0	0	
Solid-solution	UTS (MPa)	223.9	194.2	207.5	−29.7	+13.3	44.8	37.5 ± 0.1
	UTS-Error	±6.1	±4.4	±4.8	±1.7	±0.4	±0.2	
	EL (%)	16.2	10.9	12.5	−5.3	+1.6	30.2	
	El-Error	±0.3	±0.2	±0.2	±0.1	0	0	

## Data Availability

Not applicable.
